# Subsidence of callotasis zone in distraction osteogenesis after external fixator removal, measured by RSA

**DOI:** 10.3109/17453674.2010.533934

**Published:** 2010-11-26

**Authors:** Ragnhild B Gunderson, Harald Steen, Joachim Horn, Leif Pål Kristiansen

**Affiliations:** ^1^Department of Radiology; ^2^Biomechanics Laboratory; ^3^Department of Orthopaedics, Oslo University Hospital (Rikshospitalet), Oslo, Norway

## Abstract

**Background and purpose:**

In clinical practice, achieved lengthening of a callotasis zone should be maintained after the external fixator has been removed. The common understanding has been that the regenerated bone may subside. To investigate this, we used high-resolution radiostereometric analysis (RSA) with accurate measurement of the lengthening zone.

**Patients and methods:**

We assessed the longitudinal subsidence of a callotasis zone after removal of the external fixator in distraction osteogenesis in 16 patients who underwent 17 segmental lengthening operations on the tibia (n = 9) or femur (n = 8). Median lengthening was 32 (6–80) mm. RSA was performed at the end of the consolidation period before the external fixation device was removed, and this was later repeated at a median time of 11 (4–32) weeks after frame removal.

**Results:**

A minimal median longitudinal change of 0.01 (–0.28 to 0.60) mm across the lengthening zone occurred in uncomplicated cases.

**Interpretation:**

Our results indicate that no subsidence of clinical interest occurs after external frame removal.

Little is known about the subsidence of the callus in a lengthening zone after removal of the external device in distraction osteogenesis (DO). In clinical practice, it is believed to be insignificant, and the orthopedist will perform a lengthening procedure corresponding to the estimated difference in leg length. However, depending on the maturity and stiffness of the newly formed bone, gradients of subsidence are expected to occur. Thus, it is of interest to know exactly how the regenerated bone in the callotasis zone behaves after removal of the external fixator. This radiostereometric analysis (RSA) study was designed to answer this question.

## Methods

26 patients older than 10 years with tantalum spheres implanted in the bone at the operation completed a lengthening procedure by use of external distraction in our clinic, between November 2007 and October 2009. 10 patients were excluded. In 6 patients, the tantalum spheres were not properly placed to give an acceptable analysis, and in 2 patients the external frame was dynamized after the pre-removal RSA examination was performed. 1 patient was excluded because he developed a pseudarthrosis. Another patient had delayed healing with pin loosening and bone grafting, and finally had a fracture fixated with a plate and screws that interfered with free exposure of the RSA markers.

One patient had lengthening of both the femur and the tibia. Thus, 17 bone segments from 16 patients (median age 15 (10–44) years, 8 women) were included (Table). A lengthening osteotomy was performed in the proximal tibial metaphysis in 9 segments; the other 8 were distal femoral lengthenings.

**Table:** Subsidence values with time intervals between removal of the external fixator and first RSA examination, and between the two post-removal examinations

Patient	Lengthening (mm)	Obs. time 1 (weeks)	Subsidence 1 (mm) **^a^**	Obs. time 2 (weeks)	Subsidence 2 (mm) **^a^**
T1	25	24	0.05	31	–0.03
T2	31	7	–0.04		
T3	25	4	–0.05	15	–0.13
T4	55	11	–0.03		
T5	6	22	0.01		
T6	32	16	0.54	24	0.25
T7	38	10	–0.28		
T8	15	4	0.03	17	–0.06
T9	25	32 **^b^**	–0.11		
F1	34	12	0.59		
F2	80	8	0.03	70	0.27
F3	32	23	0.09		
F4	25	6	–0.13	28	0.00
F5	40	32 **^b^**	0.12		
F6	47	20	0.08		
F7	30	7	–0.12	16	–0.37
F8	60	8	–0.06	23	–0.15
Median	32	11	0.01	24	–0.05
Min	6	4	–0.28	15	–0.37
Max	80	32	0.59	70	0.27

**^a^** Negative value = shortening; positive value = lengthening.

**^b^** same patient. T: tibia; F: femur.

Before completing the operative procedure, 3–9 tantalum spheres, 1 mm in diameter, were inserted with a hand-held pistol into the bone on each side of the osteotomy through the skin incision, intending to obtain a good spread of the markers. Ideally, the spheres should be placed in the corners of a tetrahedron. All patients underwent a combined lengthening and axial correction procedure by use of the Taylor Spatial Frame (TSF).

The lengthening period depended mainly on the magnitude of the anisomelia and the age of the patient. The median degree of lengthening was 32 (6–80) mm, as controlled by successive measurements on plain radiographs that were calibrated with the known diameter of the pins with olive used in the TSF.

All patients were monitored throughout the consolidation period with radiographs, load-share measurements (Aarnes et al. 2005), and RSA registrations. The criteria for removing the fixator were based on the existence of 3 visible cortices in the callotasis zone on the radiographs (Fischgrund et al. 1994) and on load-share values of less than 10%.

Two—and in some cases 3—RSA examinations were carried out in each patient. The first RSA (baseline measurement) was performed at a median time of 1 (0–22) days before removal of the fixator, and the next RSA measurement was at least 4 weeks after removal; the median time interval was 11 (4–32) weeks. For 8 patients, we had 2 post-removal examinations. This last measurement was done after another median interval of 24 (6–70) weeks.

A standard RSA setup was used except for vertical mounting of the calibration cage (RSA Biomedical cage no. 43, 2003), including the cassettes with the 2 phosphorous plates (corresponding to X-ray films) (Figure). The beams of the 2 ceiling-mounted X-ray tubes were directed horizontally at an 80-degree angle relative to each other. These tubes were fired simultaneously during each recording. The patient was instructed to stand with the knee straight, with the affected leg at the crossing of the 2 X-ray beams and the foot in question resting on an electronic scale. If necessary, standing was supported by crutches. The weight on the affected leg was the same at all exposures and was approximately 10% of total body weight.

**Figure:**
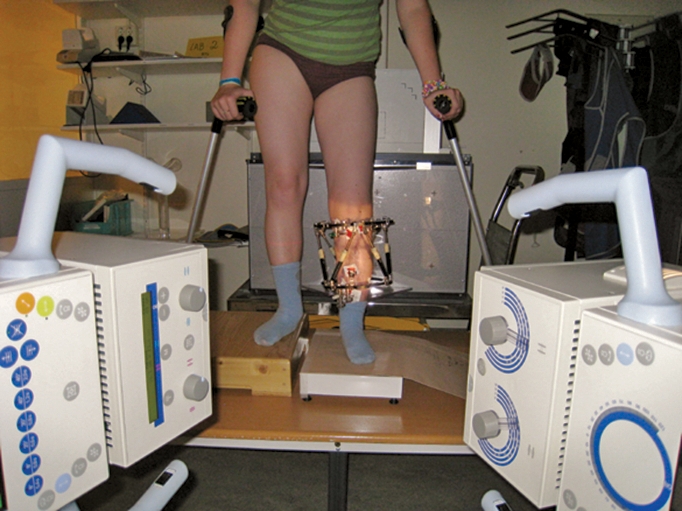
The standard examination setup with the patient in standing position and weight bearing recorded on a scale for RSA measurement of callotasis subsidence.

To reduce the risk of occlusion of the bone markers by metal, the 6 oblique struts of the TSF were usually converted into 3 vertical rods at the end of the distraction period. A single radiologist (RBG) participated at every examination to optimize the projections. The X-ray images were sent to the PACS (Picture Archiving and Communication System) and then electronically to the RSA calibration program (Um RSA Analysis, version 6.0; RSA Biomedical AB, Sweden). The distance between the 2 intact bone segments, above and below the callotasis respectively, was calculated by use of reconstruction procedures based on the fixed positions of the tantalum spheres in the calibration cage. Using the RSA software, we also calculated the change in this distance between 2 subsequent examinations. All analyses were performed by the same radiologist (RBG). Optimal analysis with 2 rigid bodies (bone segments with at least 3 valuable markers) was obtained in 14 of 25 examinations. Central values are reported as medians and dispersion as ranges. Condition numbers up to 200 (referring to the spread of the visible markers in each bone segment) (Ryd et al. 2000) and mean error values lower than 0.300 (referring to the stability of the markers) (Valstar et al. 2005) were accepted for inclusion in the analyses.

Accuracy and reproducibility of the measurements were calculated as 95% tolerance intervals according to the definitions by Ranstam et al. (2000).

The project was approved by the Regional Ethics Committee (project # 07181a 2.2007.1389). Patients (and parents of minors) had to give their written consent in order to be included.

## Results

The median subsidence between examinations before and after frame removal was 0.01 (–0.28 to 0.59) mm (the negative value indicating compression of the callotasis zone between the two events and the positive value indicating lengthening) (Table). In the 8 patients who had another post-removal examination, the median change between the 2 examinations was –0.05 (–0.37 to 0.27) mm. All rotational values along the cardinal axes in the current study were less than 1.5°. The accuracy and repeatability of the measurements were found to be ± 0.30 mm and ± 0.43 mm, respectively.

## Discussion

There have been very few reports dealing with possible changes in length of the callotasis zone after removal of an external fixator in DO. Fracture or collapse of the regenerated bone rarely occurs, but a small compression of the newly formed bone that cannot be seen on plain radiographs may be more frequent. There have been anecdotal reports of this delayed complication phenomenon (Aldegheri et al. 1989, Paley 1990). To our knowledge Shyam et al. (2009) are the only authors to have published measured values of delayed loss of length or callus subsidence in DO. They found a tibial subsidence (compression) of 4–32 mm, with about half of the 81 lengthened segments in 48 patients showing a subsidence of 1 cm or more. However, we have argued that the authors did not consider the effect of the divergence of the X-ray beam (Gunderson et al. 2010). They made direct measurements on the electronic images without calibration for the change in distance between the tibia and the film before and after removal of the ring fixator. This error has been acknowledged by the authors (Song and Shyam 2010).

Deformation of the callotasis zone during weight bearing in the early consolidation phase, as measured by RSA, has been reported previously by our group (Steen et al. 2001). In the current RSA study of subsidence after frame removal, all patients had changes of less than 0.6 mm. Some patients even showed increased lengths (within the errors of measurement). These values are an order of magnitude lower than values of clinical interest. In our setup, variations will happen due to functional conditions. Optimal results are only achieved when the film is exactly parallel to the leg and the radiation beam is directed exactly perpendicular to the bone segment in question. During standing, it is more difficult to position the leg at precisely the same angle at all events. Thus, the parallelism between the leg and the cage may differ, possibly even more than with the standard supine RSA position when dealing with hip examinations, for example. Change in parallelism will make the leg project with a little different length in the system, a known problem with all radiographic examinations. The error caused by the divergence of the radiation beam, due to the distance between the leg and the film, was eliminated in our setup by the calibration cage.

In RSA studies of hips, a condition number (CN) of up to 100 is desired (Kärrholm et al. 1997), and an upper limit of 150 is generally recommended (Valstar et al. 2005). Due to disturbing metal in the external fixator and suboptimally located markers, we chose to accept CNs of up to 200. However, in this study we only deal with translation along a single (y-) axis with minimal rotational movements. In cases where we were able to define 2 rigid bodies, rotational movements could be calculated and there were only small values (maximum 1.5°) along the two horizontal (x- and z-) axes. This indicates negligible contributions to measured movements in the vertical direction between the bone segments. With a considerable amount of rotational movement, the locations of the markers in the bone would be of critical interest. For example, a marker located anteriorly in the bone would mimic shortening (or lengthening) if there was a simultaneous rotation of significant magnitude between the bone segments (rigid bodies) in the sagittal or coronal planes. As mentioned above, we were not always able to define 2 rigid bodies (1 for each intact bone segment on each side of the callotasis). However, since no substantial rotational movement occurred it is not imperative to deal with 2 rigid bodies in this study, as 1 or 2 single markers would be expected to behave in the same way as a well-defined rigid body. Thus, we argue that it is possible to lower the demands for optimal marker positioning in this kind of study.

The RSA method itself has a high accuracy, and changes in order of 0.01 mm can be detected (Kärrholm et al. 1997). In most reports dealing with RSA, the precision in clinical settings has been found to vary between 0.15 and 0.60 mm (Steen et al. 2001, Kärrholm et al. 1997). These studies have been performed with old calibration cages and manual measurements made on scanned radiographic films. In the present work, the accuracy and precision of the measurements were estimated to be 0.30 mm and 0.43 mm, respectively. By the use of optimized conditions with the newer model of calibration cage (# 43), 2 ceiling-mounted X-ray tubes fired by 1 single button, no scanning of films, and the latest version of software, a better precision would be expected (Börlin et al. 2002, Mäkinen et al. 2004). The range of subsidence from –0.37 to 0.27 mm between the 2 post-removal examinations can be regarded as a measure of error or precision of our study. These errors are most likely due to practical and functional conditions: e.g. the geometry of the location of the spheres in the bone, the degree to which the spheres are hidden by the metal, and finally, the positioning of the patient at the moment of examination. However, all our measured values were less than the size of the error or less than 1 mm, which indicates that the subsidence of callotases after removal of the external fixator is negligible and of no clinical interest.
